# Characterization of Sub-Regional Variation in *Saccharomyces* Populations and Grape Phenolic Composition in Pinot Noir Vineyards of a Canadian Wine Region

**DOI:** 10.3389/fgene.2020.00908

**Published:** 2020-08-31

**Authors:** Elaine Cheng, Jonathan T. Martiniuk, Jonah Hamilton, Garrett McCarthy, Simone Diego Castellarin, Vivien Measday

**Affiliations:** ^1^Wine Research Centre, Faculty of Land and Food Systems, The University of British Columbia, Vancouver, BC, Canada; ^2^Department of Biology, The University of British Columbia, Kelowna, BC, Canada

**Keywords:** anthocyanin, fermentation, flavonol, flavonoid, *Saccharomyces cerevisiae*, *Saccharomyces uvarum*, *Vitis vinifera* L., wine

## Abstract

Wine is a product of grape juice fermentation by yeast. *Terroir* is a term that encompasses all environmental factors and interactions at a specific geographical site, resulting in the development of regional-specific microbial strains and grape metabolites. In this study we determine the distribution of vineyard-associated wine yeast strains and characterize the flavonoid profile of Pinot Noir grapes among 3 sub-regions in the Okanagan Valley (OV), a major wine region in British Columbia, Canada. Pinot Noir grape samples were collected from 13 vineyards among 3 sub-regions of the OV, namely Kelowna (KE), Naramata-Penticton (NP) and Oliver-Osoyoos (OO), within a week prior to the winery harvesting date in 2016 and 2017. A total of 156 spontaneous Pinot Noir fermentations were conducted and vineyard-associated *Saccharomyces* strains were isolated from fermentations that reached two-thirds sugar depletion. Using microsatellite genotyping, we identified 103 *Saccharomyces cerevisiae* strains and 9 *Saccharomyces uvarum* strains. We also identified *Saccharomyces paradoxus* in one vineyard using ITS sequencing. We developed a microsatellite database of 160 commercial *S. cerevisiae* strains to determine the identity of the isolated strains and we include the database herein. Commercial strains were widely distributed across the three sub-regions. Forty-two of our 103 *S. cerevisiae* strains were equivalent or highly similar to commercial strains whereas the remaining 61 were considered as ‘unknown’ strains. Two *S. uvarum* strains were previously isolated in other OV studies and none matched the *S. uvarum* commercial strain BMV58. *S. cerevisiae* population structure was driven by sub-region, although *S. cerevisiae* populations did not differ significantly across vintages. *S. uvarum* and *S. paradoxus* were only identified in the 2017 vintage, demonstrating dynamic wine yeast populations between vintages. We found that the flavonoid profile of Pinot Noir grapes from the same 13 vineyards was also affected by sub-regional *terroir*. The anthocyanin content was lower and the proportion of methoxylated anthocyanins and flavonols was higher in Pinot Noir grapes from OO, the warmer sub-region as compared to KE, the cooler sub-region. Our study demonstrates that both yeast populations and metabolites associated with the Pinot Noir variety have sub-regional variation within a viticultural area.

## Introduction

The characteristics of a wine region are believed to influence the sensory profiles of wines made in these regions, a concept referred to as *terroir* (van Leeuwen and Seguin, 2006). These characteristics include abiotic factors such as climate, topography and topology as well as biotic factors such as soil, fruit and vineyard microbiota. While regional abiotic factors have a more obvious impact on the differentiation of wine characteristics, the regional composition and structure of microbial communities has been correlated with differences in wine chemical and sensory profiles (Knight et al., 2015; Bokulich et al., 2016). In particular, the composition of wine grape phenolic compounds, which are important for red wine quality, can be influenced by *terroir*. Anthocyanins determine the color of the red grape and wine (Gould and Lister, 2005). Tannins confer astringency and bitter sensorial notes to grapes and wines, and provide texture to red wines (Flamini and Traldi, 2010). Flavonols influence wine quality by forming non-covalent interactions with anthocyanin molecules. The interaction of flavonols with anthocyanins results in color intensity enhancement of grape skins (Pollastri and Tattini, 2011; Trouillas et al., 2016). Abiotic factors such as light, temperature, and water availability strongly affect grape phenolic compounds (Castellarin et al., 2007; Mori et al., 2007; Cohen et al., 2008; Matus et al., 2009; Savoi et al., 2017). Arguably, the soil and the above abiotic factors, are the major components of the *terroir* that affect the phenolic composition of the grapes and wines (van Leeuwen, 2010; Willwerth et al., 2010; Ferndandez-Marin et al., 2013; Artem et al., 2016; Del-Castillo-Alonso et al., 2016).

The Okanagan Valley (OV) is a major wine region of Canada. The narrow valley spans from the United States border with Washington State to approximately 250 km north and is marked by several lakes. The OV climate is considered cool and arid (Senese et al., 2012). Given its great latitudinal range, the OV has many microclimates and diverse soil types, lending itself to the production of premium wines made from many grape cultivars (Bowen et al., 2005). While the majority of red wine grapes are grown in the Oliver-Osoyoos (OO) area at the south end of the valley (containing Canada’s only semi-desert), Pinot Noir is commonly grown in all areas of OV. However, more acres of Pinot Noir are planted in the Naramata-Penticton (NP, central) and more northern Kelowna (KE) sub-regions. Pinot Noir is an early ripening vine and therefore cool climate regions provide ideal viticulture sites to prevent pre-maturation of grapes that may cause loss of acidy and aroma. Little information exists on the microbial populations in OV vineyards and the variation of phenolic accumulation in grape berries. Knowledge of the regional variation in Pinot Noir flavonoid grape composition (anthocyanins, tannins, flavonols) will constitute baseline information for investigating the impact of OV *terroir* on Pinot Noir wine quality.

*Saccharomyces* species are essential in winemaking both for their roles in alcoholic fermentation and for their influence on wine organoleptic profiles through the production or release of volatile compounds (Fleet, 2003; Styger et al., 2011; Cordente et al., 2012). *Saccharomyces cerevisiae* is by far most common in wine fermentations, while *S. uvarum* may also occur in mixed populations with *S. cerevisiae* or as the dominant yeast in white wine fermentations (Demuyter et al., 2004; Tosi et al., 2009; Masneuf-Pomarede et al., 2010). There are two strategies of wine fermentation: inoculated and spontaneous. Inoculated fermentation is initiated by the addition of an industrial *Saccharomyces* strain to grape must*;* by our estimation, over 150 industrial *S. cerevisiae* strains and one *S. uvarum* strain are available for winemaking purposes. The use of industrial starter cultures, of which a diverse array of strains is available, is popular amongst winemakers due to the reliability of a rapid fermentation that completes in a timely manner. Spontaneous fermentation, in contrast, is carried out by yeasts present on grape and winery surfaces that include various fermentative species from several yeast genera. Spontaneous fermentations may be initiated by a variety of weakly fermentative yeast species but by the end of fermentation are typically dominated by one or often multiple strains of *S. cerevisiae* and/or *S. uvarum* (Pretorius, 2000; Varela and Borneman, 2017). Wines made by spontaneous fermentation may have more complex sensory profiles than those fermented by single strain inocula due to the diverse metabolic activity of multiple yeast species and strains (Jolly et al., 2014).

*Saccharomyces cerevisiae* wine strains across global wine regions have demonstrated close genetic relatedness; they cannot be satisfactorily differentiated by geographical origin but are distinct from strains in other ecological niches (Legras et al., 2007; Liti et al., 2009; Schacherer et al., 2009; Almeida et al., 2015; Peter et al., 2018). Nevertheless, *S. cerevisiae* population structure has been identified on a smaller scale across various wine regions in France, Portugal, New Zealand and Italy spanning tens to hundreds of kilometers (Gayevskiy and Goddard, 2012; Schuller et al., 2012; Knight and Goddard, 2015; Borlin et al., 2016; Rantsiou et al., 2017). A factor confounding analysis of regional *S. cerevisiae* population structure is the dissemination of commercial wine strains into the winery and surrounding environment. Commercial *S. cerevisiae* strains may be highly abundant or dominate spontaneous fermentations in wineries using commercial strains (Beltran et al., 2002; Hall et al., 2011; Tello et al., 2011; Scholl et al., 2016). However, indigenous strains can dominate spontaneous fermentations and outcompete commercial strains when co-inoculated (Tello et al., 2011; Capece et al., 2019). In large-scale surveys of *S. cerevisiae* populations in vineyards and wineries across multiple wine regions, commercial strains were found to represent a very low proportion of the total yeast population (Gayevskiy and Goddard, 2012; Schuller et al., 2012; Knight and Goddard, 2015). However, widespread dissemination of commercial strains was detected across three Italian wine regions (Viel et al., 2017). The presence of commercial yeast strains was also found to be a driver of population structure in a small-scale vineyard and winery (Martiniuk et al., 2016). In all cases, identification of commercial wine isolates is dependent on access to a database containing the genetic strain profile of each commercial strain.

In contrast to *S. cerevisiae*, less information exists on the population structure and genetic diversity of *S. uvarum*. Sequence analysis of 54 *S. uvarum* strains revealed three clades including a holoarctic clade comprised of natural isolates and wine-making strains from North America, Europe and the Far East (Almeida et al., 2014). Microsatellite analysis of a larger group of *S. uvarum* strains consisting mostly of European wine and cider isolates did not reveal a strong association between genetic relatedness and region of origin (Masneuf-Pomarede et al., 2016). Another clear difference between *S. cerevisiae* and *S. uvarum* winery and vineyard isolates is that a much higher percentage of *S. uvarum* wine strains are homozygous, suggesting that the species is in-bred (Zhang et al., 2015; Masneuf-Pomarede et al., 2016). However, two recent studies identified a highly diverse heterogenous population of *S. uvarum* in spontaneous Chardonnay fermentations from an OV winery in two separate vintages (Morgan et al., 2019; McCarthy et al., Unpublished). A subset of the OV *S. uvarum* strains are genetically distinct from global *S. uvarum* strains and have a higher degree of heterozygosity based on microsatellite analyses (Morgan et al., 2019).

Herein, we elucidate the regional population structure of vineyard-associated *Saccharomyces* strains among three winemaking sub-regions of the Okanagan Valley (KE, NP, and OO) over two vintages (2016–2017), and compile a database of over 150 commercial *S. cerevisiae* strains to profile commercial yeast dissemination across the region. We also denote the different flavonol and anthocyanin profiles and seed tannin levels of Pinot Noir grapes between the three sub-regions in the 2017 vintage. Our work is the first study looking at the regional-specificity of Pinot Noir grapes in the OV, British Columbia, Canada.

## Materials and Methods

### Experimental Design

**FIGURE 1:**
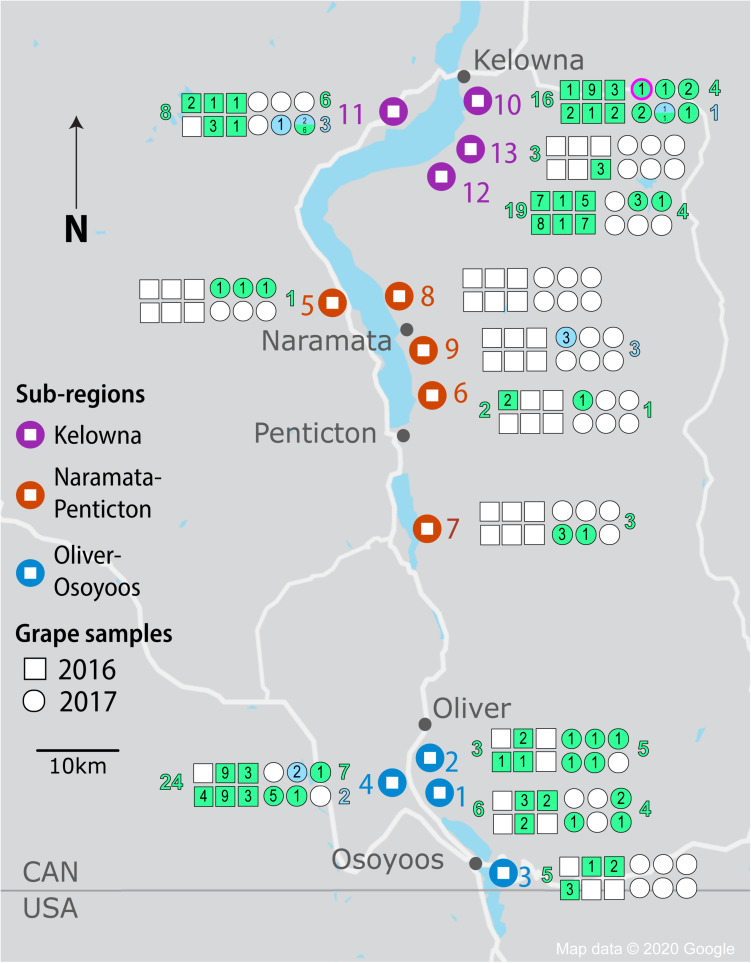
Isolation of *Saccharomyces* yeasts by sample, vineyard, sub-region and vintage from the OV wine region. Vineyard locations are denoted by large markers and colored by region (KE-purple, NP-orange, and OO-blue). Squares and circles represent each of 6 spontaneous fermentations conducted for each vineyard in 2016 and 2017, respectively. Shaded squares and circles indicate fermentations that reached two-thirds sugar depletion; green shading indicates *Saccharomyces cerevisiae* strains while blue shading indicates *Saccharomyces uvarum* strains. The number of strains isolated from a spontaneous fermentation is noted within each square or circle. The number of *S. cerevisiae* and *S. uvarum* strains isolated in total from each vineyard are listed in green and blue, respectively (to the left of the vineyard for 2016 fermentations and to the right of the vineyard for 2017 fermentations). The circle outlined in fuschia for vineyard #10 denotes the fermentation from which *S. paradoxus* was isolated.

In autumn 2016 and 2017, we aseptically harvested healthy grape clusters from 13 Pinot Noir vineyards from the OV within a week prior to the winery harvesting date (Figure 1). The vineyards spanned a 100 km distance from north to south and included three OV sub-regions – KE in the north, NP in the center, and OO in the south. In total, 4 KE, 5 NP and 4 OO Pinot Noir vineyards were sampled. Weather data were retrieved from three weather stations, namely “Oliver STP,” “Penticton A,” and “Kelowna UBCO” (Government of Canada, 2020). Growing degree days (GDD) were calculated as base 10°C degree-days from 1st April to 31 October 2017 and cumulative precipitations for the same period was identified (Amerine and Winkler, 1944).

### Grape Sampling and Processing

A ∼0.25 hectare area of each Pinot Noir vineyard was selected for sampling with outer vineyard rows and the first 6 m of each row excluded. Each area was sub-divided into thirty-two 18 m sections of two rows; six sections were randomly selected for sampling. Each sample consisted of 30 grape clusters aseptically harvested within each section. All samples were transported directly to the lab on ice and processed within 24 h of harvest. Each sample was manually crushed for 15 min; 500 mL of juice (excluding skins and seeds) was transferred from each sample to a sterile airlock-sealed vessel and fermented aseptically, for a total of 78 fermentations (six per vineyard) for each vintage. Fermentations were conducted at 25°C and sampled at two-thirds sugar depletion (as determined by weight loss). Fermentations that did not reach two-thirds sugar depletion within 40 days were excluded from further analysis.

In 2017, 4 of the 6 sections selected for yeast isolation in each vineyard were randomly selected for grape composition analyses. Forty berries per section were randomly picked by hand for total soluble solids (TSS) analyses. Another 40 berries per replicate were randomly harvested with scissors for flavonoid analysis by cutting off the berry at the pedicel level to avoid any damage that could create oxidation to the berry. The grape samples were taken to the laboratory on dry ice, where they were stored in a −80°C freezer until processing.

### *Saccharomyces* Isolation and DNA Extraction

At two-thirds sugar depletion, fermentation samples were serially diluted in 0.1% peptone and plated in duplicate on yeast extract-peptone-dextrose (YPD) agar plates containing 0.015% biphenyl and 0.01% chloramphenicol to inhibit mold and bacterial growth, respectively (Singleton and Sainsbury, 1987; Luck and Jager, 1997). For each fermentation sampled, up to 48 *Saccharomyces* colonies were isolated when possible from a plate containing 30–300 colonies. Suspected *Saccharomyces* colonies were confirmed by plating on Wallerstein nutrient agar, which differentiates *Saccharomyces* and non-*Saccharomyces* yeasts based on colony color, and cross-verified by negative growth on lysine agar (Heard and Fleet, 1986; Pallman et al., 2001). All isolates were arrayed in 96-well plates and frozen at −80°C. Yeast DNA was extracted as previously described and diluted to 25 μg/μl in sterile 10 mM Tris pH 8.0 (Martiniuk et al., 2016).

### Differentiation of *S. cerevisiae* and *S. uvarum* Isolates

*Saccharomyces cerevisiae* and *S. uvarum* were differentiated based on polymorphism at the *MET2* locus using restriction fragment length polymorphism (RFLP)-polymerase chain reaction (PCR) (Masneuf et al., 1996). The diluted DNA (1 μl) was added into 19 μl of *MET2* PCR master mix [20% 5× dNTPs, 10% 10× BioBasic Taq Buffer, 10% MgSO4 (20 mM), 0.6% *MET2* forward primer (100 μM), 0.6% *MET2* reverse primer (100 μM), 0.5% BioBasic Taq Polymerase]. Amplification was performed under the following conditions: initial denaturation step of 5 min at 94°C, 35 cycles of 30 s at 94°C, 45 s at 50°C and 1 min at 72°C, and a final elongation step of 10 min at 72°C. The PCR products were digested separately by *EcoRI* and *PstI* at 37°C for 30 min, then analyzed by electrophoresis on a 1.2% agarose gel. *EcoRI* digests only the *MET2* gene of *S. cerevisiae* whereas *PstI* digests only the *MET2* gene of *S. uvarum.*

### Identification of *Saccharomyces paradoxus*

For DNA from isolates with *MET2* that could not be digested with *EcoRI* or *Pst1*, internal transcribed spacer (ITS)-PCR was performed to identify the species. The diluted DNA (1 μl) was added into 19 μl of PCR master mix [20% 5× dNTPs, 20% 5× Phusion HF buffer, 0.5% ITS1 primer (100 μM), 0.5% ITS4 primer (100 μM), 1% Phusion Taq Polymerase] (Op, De Beeck et al., 2014). ITS amplification was performed under the following conditions: initial denaturation step of 2 min at 98°C, 35 cycles of 10 s at 98°C, 30 s at 62°C, 30 s at 72°C, and a final elongation step of 10 min at 72°C. The ITS PCR products were cleaned by the E.Z.N.A Cycle Pure Kit and sent to Genewiz for Sanger sequencing. The sequencing data were analyzed by BLAST to identify the species.

### Commercial *S. cerevisiae* Collection Compilation

One hundred and sixty commercial *S. cerevisiae* strains were generously donated or purchased from various companies and wineries and from the lab of Dr. Daniel M. Durall, University of British Columbia, Canada. Strains received on slants were streaked for single colonies on YPD agar, while active dry yeasts were rehydrated for 10 min in water and streaked for single colonies on YPD agar. Certain commercial strains were sourced from multiple locations; in cases where genotypes from the same strain differed between sources, all MLGs were included in the database (Supplementary Table S1).

### Microsatellite Analysis

Isolated and commercial *S. cerevisiae* strains were genotyped using ten short tandem repeat (STR) loci selected from previous studies (Supplementary Table S2) (Legras et al., 2005; Richards et al., 2009). These loci were amplified according to (Martiniuk et al., 2016). For *S. uvarum*, we selected eleven STR loci that were previously identified (Zhang et al., 2015; Masneuf-Pomarede et al., 2016) (Supplementary Table S3). Each STR amplification reaction contained 5 μl of Qiagen Multiplex PCR Master Mix, 4 μl of primer mix, and 1 μl of diluted DNA. Primer sequences for *S. cerevisiae* and *S. uvarum* microsatellite amplification are provided in Supplementary Tables S4, S5, respectively. The PCR was carried out with an initial denaturation step of 5 min at 97°C; 34 cycles of 30 s at 95°C, 60 s at 54°C, and 120 s at 72°C; and a final elongation step of 10 min at 72°C. Microsatellite amplicons were analyzed at the UBC Sequencing and Bioinformatics Consortium on an AB3730 DNA Analyzer. GeneMapper software was used to generate a multi-locus genotype (MLG) for each amplicon profile. Each unique MLG was considered as an individual strain.

### *Saccharomyces* Population Data Analysis

*Saccharomyces* MLG counts and genetic distance calculations were performed in R v.3.5.3 using the poppr package v.2.6.0 (Kamvar et al., 2014; Kamvar et al., 2015). *S. cerevisiae* isolate MLGs were compared against our *S. cerevisiae* commercial strain collection using a custom R script based on the genetic relatedness measure Bruvo Distance (BD) (Bruvo et al., 2004). Isolate MLGs ≤ 0.25 BD from the closest commercial strain relative were classified as commercial strains whereas the remainder were classified as unknown. *S. uvarum* isolate MLGs were compared against a database of 20 *S. uvarum* strains previously isolated from around the world, 10 *S. uvarum* strains previously isolated in OV wineries and 1 *S. uvarum* commercial strain, BMV 58 (Supplementary Table S6). Venn diagrams were created in Jvenn to visualize the number of strains isolated in each sub-region and each vintage (Bardou et al., 2014). Analysis of molecular variance (AMOVA) was performed in poppr and Bayesian clustering of MLGs in InStruct (Gao et al., 2007). Subpopulation membership in InStruct was determined using the admixture model with a burn-in of 50,000 iterations, 200,000 iterations per chain with 5 chains per cluster, or *K*, from *K* = 3 to *K* = 20. The Instruct analysis was narrowed to *K* = 7 through 12 with 5 chains of 1,000,000 iterations per K and a burn-in of 100,000. The optimal K or number of sub-populations was determined using the Deviance Information Criterion method (Gao et al., 2011). The InStruct output was aligned in CLUMPP using the LargeKGreedy algorithm (Jakobsson and Rosenberg, 2007) and visualized in DISTRUCT (Rosenberg, 2004). The correlation between InStruct inferred population structure and sub-regions or sub-populations of interest was evaluated in ObStruct (Gayevskiy et al., 2014). Phylogenetic networks were produced in SplitsTree 4.14.6 using the Neighbor-Net algorithm (Bryant and Moulton, 2004; Huson and Bryant, 2006).

### Grape Compositional Analysis

The berries collected for TSS analysis were weighed and squeezed, and the juice was analyzed with a digital refractometer (Sper Scientific 300017). For flavonoid analysis, the pedicels were removed from the berry samples before the weight was taken. The berries were carefully dissected by a scalpel to separate the skin and seed materials. Skins and seeds were weighed and kept frozen in liquid nitrogen before being ground into a fine powder using mortars and pestles. The fine powder samples of skins and seeds were stored in a −80°C freezer prior to flavonoid analyses.

Anthocyanin and flavonol extractions were completed as described (Downey and Rochfort, 2008). The extraction was performed twice on the skin fine powder (0.180 g samples with 1.8 g solvent in each extraction), then the solution was filtered by a 3-mL Luer-Lok Tip syringe coupled with a 0.22 mm × 13 mm PVDF filter, and diluted 10-fold with the extraction solvent. Diluted extracts were injected into an Agilent 1100 Series LC/DAD/MSD Trap XCT Plus System equipped with an Agilent ZORBAX SB-C18 Column (1.8 μm, 4.6 × 50 mm). The mobile phases were composed of a solvent A (water with 2% formic acid) and a solvent B (acetonitrile with 2% formic acid). The binary solvent gradient for the liquid chromatographic separation was achieved as followed: 0 min, 5% solvent B; 6 min, 20% solvent B; 9 min, 80% solvent B; 10 min, 90% solvent B; 11 min, 5% solvent B. The analysis was run at a flow rate of 1.20 mL/min at a constant temperature of 67°C. Anthocyanins were examined at 520 nm. Flavonols were examined by the mass spectrometer because co-elution occurred at 353 nm. Compound identification was conducted by (i) comparing their retention times with those of authentic standards (3-*O*-glucosides of cyanidin, peonidin, delphinidin, petunidin, and malvidin, (ii) matching the mass spectra of identified peaks with anthocyanin and flavonol compounds retrieved from published papers, and (iii) comparing their elution order (Mazza et al., 1999; Garcia-Beneytez et al., 2003; Castillo-Munoz et al., 2007; Downey and Rochfort, 2008). Anthocyanin and flavonol concentrations were reported in malvidin 3-glucoside and quercetin-3-glucoside equivalents, respectively, and expressed as μg/berry and μg/g of berry fresh weight.

For the extraction of skin and seed tannins, 0.18 g of berry skins or seeds were added to 1.8 mL of acetone/water solution (70/30) and shaken gently for 24 h. The sample was centrifuged (10 min at 14,000 *g*), then 1 mL of supernatant was removed into a new 2 mL-micro tube, and the acetone was evaporated via 1 h of speed vacuum. The residual aqueous extract was adjusted to 1 mL with deionised water. After this, tannins were measured by the protein precipitation assay (Harbertson et al., 2002). Skin and seed tannins were expressed as mg/berry and mg/g of berry fresh weight. Measurement of tannins was carried out in duplicate from each sample, and the two values obtained were averaged.

The general berry and flavonoid data are presented as mean ± standard error and analyzed by an ANOVA test where the effect of the sub-regions (*n* = 3) on the various parameters was assessed with vineyards (*n* = 13) considered as nested factors within sub-regions. Different letters indicate significant differences (*p* < 0.05) between sub-regions according to a Tukey’s HSD test. Statistical analyses were performed using JMP 14 (Statistical Discovery^TM^ from SAS Institute Inc.).

## Results

### Isolation of *Saccharomyces* Strains From Pinot Noir Spontaneous Fermentations in Three OV Sub-Regions

We chose to isolate *Saccharomyces* from Pinot Noir spontaneous fermentations once two-thirds of the sugar was depleted to identify strains that were vigorous during active fermentation. Of the 78 spontaneous fermentations conducted in each vintage, 33 (42%) in 2016 and 29 (37%) in 2017 reached two-thirds sugar depletion (Figure 1 and Supplementary Table S7). The number of fermentations reaching two-thirds sugar depletion varied substantially among sub-regions and between vintages. Even though NP had the fewest spontaneous fermentations reaching two-thirds sugar depletion among the three sub-regions in both vintages, NP had many more successful fermentations in 2017 than 2016 (7 out of 30 compared to 1 out of 30, respectively). In contrast, both OO and KE had fewer successful fermentations in the 2017 vintage than 2016 vintage (Figure 1 and Supplementary Table S7). In 2016, OO had 14 out of 24 fermentations reach two-thirds sugar depletion (58%) compared to 12 out of 24 (50%) in 2017. Likewise, KE had 18 out of 24 (75%) fermentations reach two-thirds sugar depletion in 2016 compared to 10 out of 24 (42%) in 2017. In both vintages, the exact same vineyards and where possible, the same locations within the vineyard were sampled.

**FIGURE 2:**
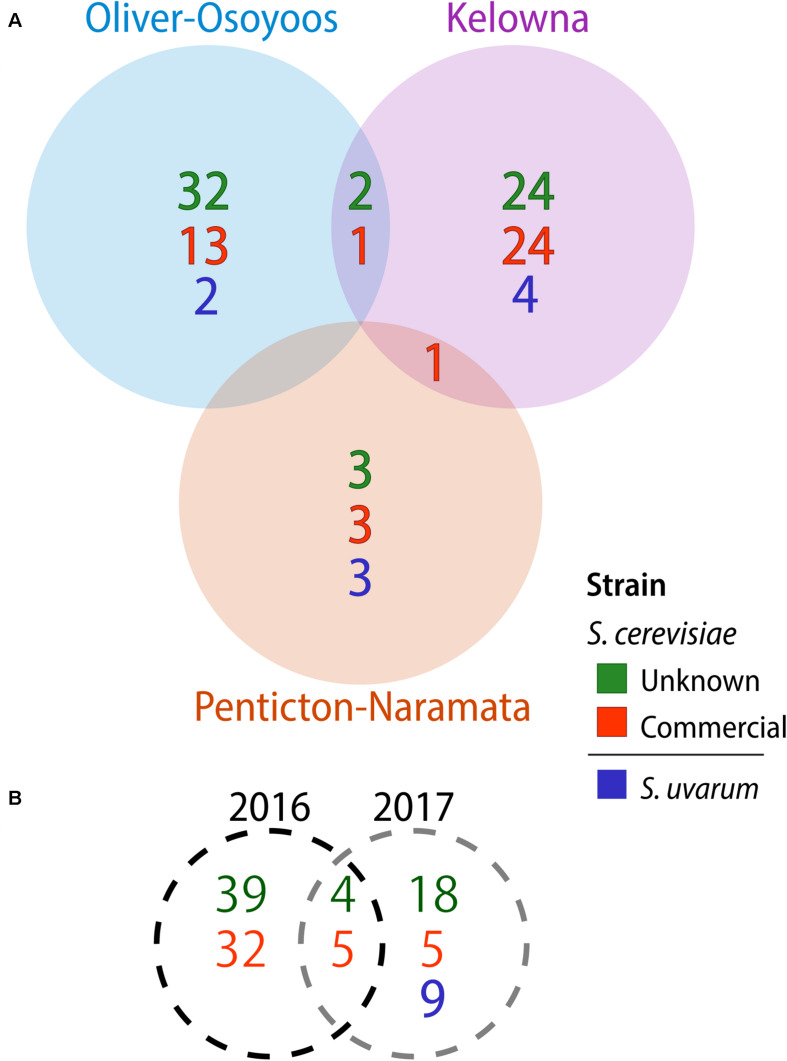
*Saccharomyces cerevisiae* and *S. uvarum* strains isolated by **(A)** OV sub-region and **(B)** vintage. Commercial *S. cerevisiae* strains are in red, unknown strains in green, while *S. uvarum* strains are blue.

A total of 1,544 *Saccharomyces* colonies were isolated in 2016 compared to 1,368 in 2017. Only *S. cerevisiae* was isolated in 2016. In contrast, in 2017 three *Saccharomyces* species were identified: 1,176 isolates of *S. cerevisiae*, 164 of *S. uvarum* and 28 of *Saccharomyces paradoxus*. Both *S. cerevisiae* and *S. uvarum* were identified in all three OV sub-regions, while *S. paradoxus* was only isolated from KE vineyard #10 (Figure 1). We used microsatellite analysis of 10 *S. cerevisiae* STR loci and 11 *S. uvarum* STR loci to genotype our *Saccharomyces* isolates whereas no genotyping was conducted on *S. paradoxus.* A total of 103 *S. cerevisiae* MLGs and 9 *S. uvarum* MLGs were identified in this study (Supplementary Tables S8, S9, respectively). Each MLG is considered to be an individual strain and will be referred to as ‘strain’ or “MLG” from now on. The distribution of strains across vintages, sub-regions, vineyards and between replicate spontaneous fermentations was highly heterogeneous (Figure 1). As few as one and as many as nine *Saccharomyces* strains were isolated from a single spontaneous fermentation that reached two-thirds sugar depletion. We compared our isolated *S. cerevisiae* strains against a database that we generated of 160 commercial *S. cerevisiae* strains used for wine (150 strains), beer (8 strains) and spirit (2 strains) production (Supplementary Table S1). Of the 160 commercial strains profiled in this study, only 125 MLGs were generated, indicating a high degree of redundancy between commercially available strains. Out of the 103 vineyard isolate strains, 42 were the equivalent to, or highly similar to, commercial strain isolates (≤0.25 BD from closest commercial strain relative) whereas 61 strains were different from commercial strains and will henceforth be referred to as ‘unknown’ strains. A comparison of the *S. cerevisiae* strains isolated across vintages and OV sub-regions revealed that very few strains were isolated from both vintages or multiple sub-regions (Figure 2). Three *S. cerevisiae* strains were shared between OO and KE, one between NP and KE and none between OO and NP (Figure 2A). Only 9 *S. cerevisiae* strains were identified in both 2016 and 2017 whereas 71 strains were identified solely in the 2016 vintage and 23 strains solely in the 2017 vintage (Figure 2B). While a greater number of unknown *S. cerevisiae* strains than commercial were isolated in each vintage, fewer unknown strains (4) than commercial strains (5) were shared between vintages (Figure 2B). Across sub-regions in both vintages, 24 unknown strains were isolated only from KE, 32 only from OO and 3 only from NP (Figure 2A). OO had the highest proportion of unknown strains (68%).

None of the 9 *S. uvarum* MLGs identified in this study were shared between sub-regions (Figure 2). There were 4 *S. uvarum* strains isolated from KE, 3 from NP, and 2 from OO (Figure 2A). None of the 9 *S. uvarum* MLGs were equivalent to the commercial *S. uvarum* commercial strain, BMV58 (Lallemand). However, 2 of our *S. uvarum* strains had been isolated in previous OV studies [(Morgan et al., 2019; McCarthy et al., Unpublished) and unpublished data from this group].

### *Saccharomyces* Population Structure

#### Saccharomyces cerevisiae

**FIGURE 3:**
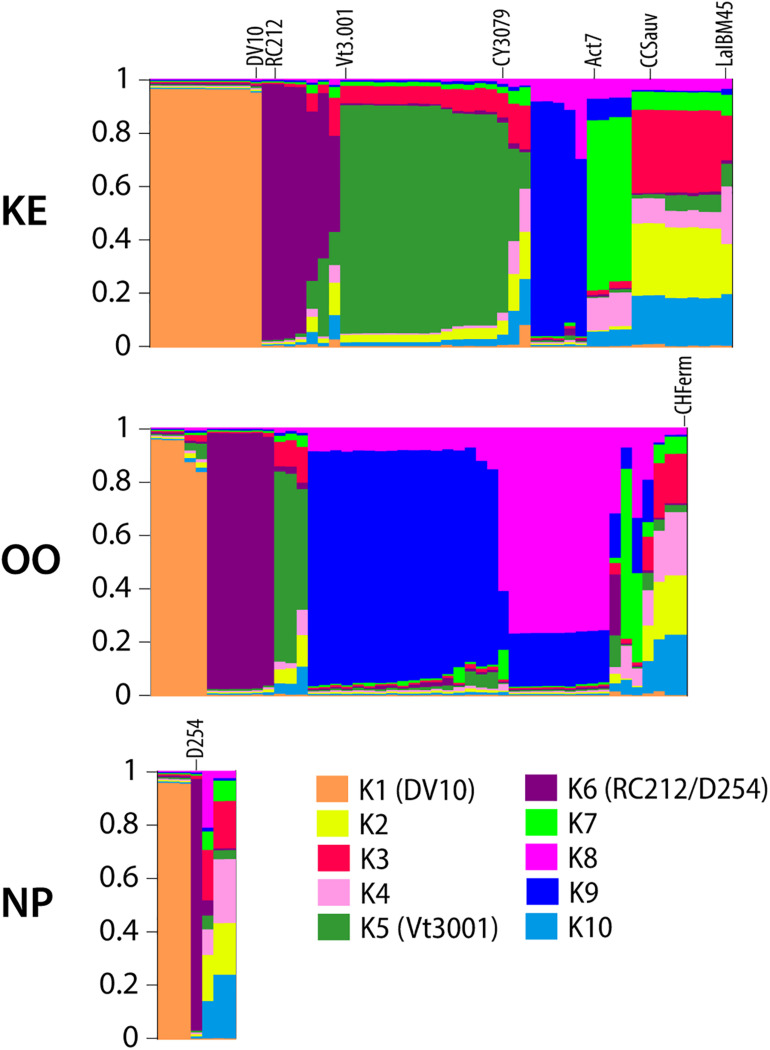
DISTRUCT plots of inferred ancestry profiles of *S. cerevisiae* strains isolated from KE, OO, and NP sub-regions. Each column represents an ancestry profile for an individual MLG. Each color corresponds to one of ten inferred subpopulations as noted in the legend. The proportion of each color in a column represents the proportion (membership coefficient) of the MLG’s ancestry profile assigned to that subpopulation as measured on the *y*-axis of each plot. Selected commercial isolate MLG ancestry profiles are labeled on the plot, and subpopulations containing commercial MLGs with membership coefficients >80% are labeled in the legend with the strain name in parentheses.

We conducted hierarchical AMOVAs on *S. cerevisiae* MLGs to elucidate strain population structure across vintages and between sub-regions, both with and without commercial MLGs included. Separate AMOVAs were conducted using vintage and sub-region as factors, and NP strains were excluded from this analysis due to small sample size (Table 1). In both analyses, within-vineyard strain populations were most variable. While very few strains were shared between vintages, AMOVA identified no significant difference between 2016 and 2017 strain populations whether or not commercial strains were included (−1.44%, *p* = 0.6415; 0.51, *p* = 0.2563), indicating that the populations in each vintage were genetically similar. In contrast, significant variation was found between KE and OO sub-regional populations with commercial strains included (5.44%, *p* = 0.0278). Notably, the variation nearly doubled when commercial strains were removed from the analysis (10.40%, *p* < 0.0295), indicating stronger spatial population structure of unknown strains. We further evaluated *S. cerevisiae* strain population structure using InStruct, a clustering software optimized for inbred or clonal populations like *S. cerevisiae* that assigns individuals in a population to a given number of statistically determined subpopulations (Gao et al., 2007, 2011). Instruct analysis identified 10 subpopulations in the *S. cerevisiae* MLG dataset (K1–K10) and ancestry profiles for each MLG by sub-region were visualized using Distruct (Figure 3). Profiles containing >80% of a particular color indicate MLGs belonging to a single subpopulation, while profiles consisting of multiple colors indicate admixed or interbred MLGs. Subpopulations K1, K5, and K6 correspond to *S. cerevisiae* commercial MLGs DV10, Vt3.001/CY3079, and RC212/D254, respectively, while other commercial strains are represented by admixture of two or more subpopulations (Figure 3). For example, LalBM45 in KE has a membership coefficient of ∼0.2 in each of K2, K4, and K10 (Figure 3). Two subpopulations, K8 and K9, are not associated with any commercial strains; these are most prevalent in the OO populations (Figure 3). InStruct inferred *S. cerevisiae* population structure is significantly correlated with sub-region (*R*^2^ = 0.13, *p* < 0.0001) as determined by ObStruct, with OO populations contributing most to sub-region as a driver of this structure (Supplementary Table S10). Interestingly, subpopulation K9 is also a strong driver of population structure (*R*^2^ = 0.06 when K9 is removed), which agrees with the increased differentiation between sub-regions when commercial strains were removed (Table 1).

**TABLE 1:** AMOVA of *S. cerevisiae* MLGs isolated in this study by vintage and sub-region, with and without commercial MLGs.

		*w/C MLGs*			*w/o C MLGs*		
		
Factor	Hierarchical level	Variation (%)	Φ	*P*	Variation (%)	Φ	*P*
Vintage	Between vintages	−1.44	−0.01	0.6415	0.51	0.01	0.2563
	Vineyards within vintages	16.71	0.16	<0.0001	21.75	0.22	<0.0001
	Within vineyards	51.35	0.61	<0.0001	59.28	0.76	<0.0001
	Within samples	33.38	0.67	<0.0001	18.46	0.81	<0.0001
Sub-region	Between sub-regions	5.44	0.05	0.0278	10.40	0.10	0.0295
	Vineyards within sub-regions	8.82	0.09	<0.0001	7.94	0.09	0.0008
	Within vineyards	53.44	0.62	<0.0001	63.58	0.78	<0.0001
	Within samples	32.28	0.68	<0.0001	18.08	0.82	<0.0001

*Significance was determined from 9999 permutations. PN strains were excluded from this analysis due to small sample size. w/C MLGs, with commercial strains isolated from this study; w/o C MLGs, without commercial strains.*

**FIGURE 4:**
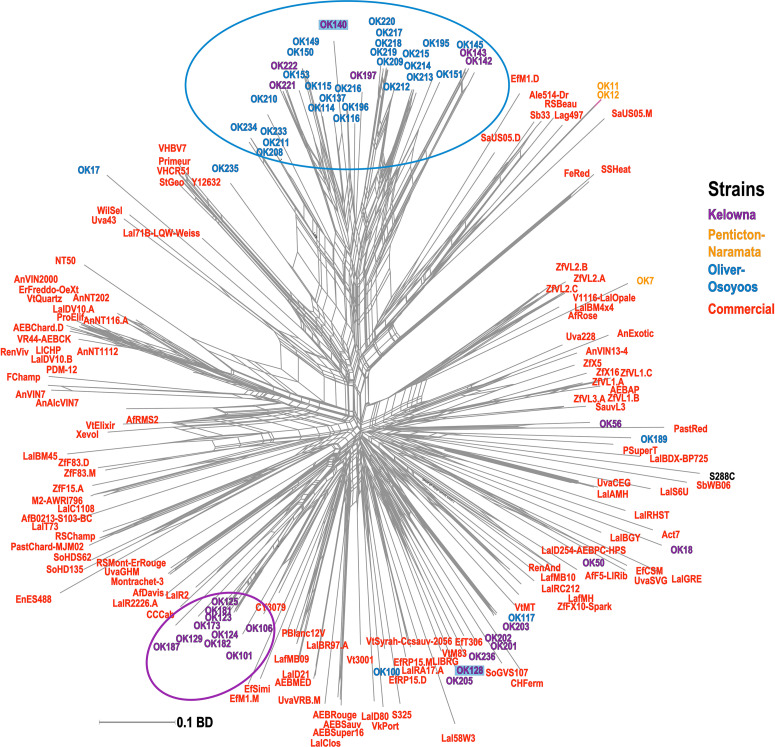
Phylogenetic network of unknown and commercial *S. cerevisiae* MLGs. The phylogenetic network was built with the Neighbor-Net algorithm using a pairwise BD matrix. Unknown strains are labeled as OK and are colored by sub-region of origin; strains isolated from multiple sub-regions are highlighted. Commercial MLGs from the commercial strain collection are in red; the *S. cerevisiae* reference lab strain S288C is in black. Distinct sub-regional clusters of strains are circled.

A phylogenetic network was constructed using a BD matrix to visualize the genetic relationships between unknown and commercial *S. cerevisiae* strains (Figure 4). While some of the strains classified as unknown appear related to commercial strains, two sub-regional clusters of unknown *S. cerevisiae* strains from OO (blue circle) and KE (purple circle) were identified. Thus, most of the unknown strains isolated from OO and KE were more genetically related to strains isolated from the same sub-region as compared to strains between sub-regions or other commercial strains (Figure 4). Additionally, many of these strains have >60% membership in the two InStruct subpopulations not associated with commercial MLGs (K8, K9, Figure 3). It should be noted that some *S. cerevisiae* strains isolated from KE were closely genetically associated with strains in the OO cluster (e.g., OK221 and OK222). In addition, OK128 and OK140 were isolated in both KE and OO (Figure 4). Unexpectedly, we found that two unknown *S. cerevisiae* strains isolated from NP (OK11 and OK12) were genetically closely associated with beer strains (Figure 4).

#### Saccharomyces uvarum

**FIGURE 5:**
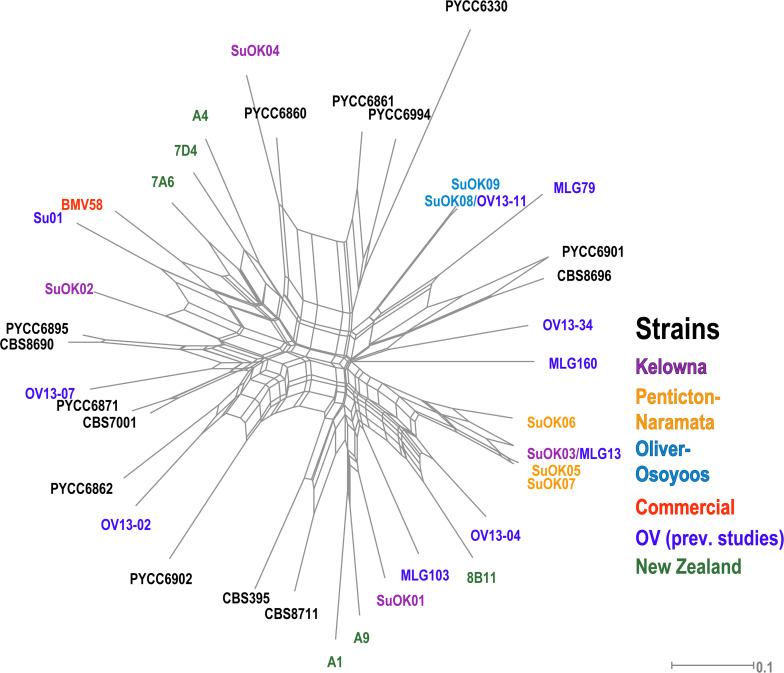
Phylogenetic network of *S. uvarum* strains. The phylogenetic relationship was established by the Neighbor-Net algorithm based on BD. Newly discovered strains are labeled as “SuOK” and colored according to the region of isolation. SuOK08 and SuOK03 are indicated with strains isolated from the OV in previous studies (OV13-11 and MLG13, respectively, Supplementary Table S6). Green strains were isolated from a study in New Zealand (Zhang et al., 2015), the commercial *S. uvarum* strain BMV58 is indicated in red and black colored strains are from CBS and PYCC databases (Supplementary Table S6).

Only 9 MLGs were identified from all 164 *S. uvarum* isolates so AMOVA could not be performed on the *S. uvarum* dataset due to the low number of strains isolated. We compared our *S. uvarum* MLGs to 31 *S. uvarum* strains previously isolated from locations in British Columbia (OV wineries, Hornby Island) and other regions worldwide and displayed the data using a phylogenetic network (Figure 5 and Supplementary Table S6). One *S. uvarum* MLG from OO (SuOK08) was identical to a strain isolated from a previously unpublished OV study that we conducted with Pinot Gris grapes and named OV13-11. Another *S. uvarum* MLG from KE (SuOK03) was identical to a strain isolated from OV spontaneous Chardonnay fermentations and named MLG13 (McCarthy et al., Unpublished). One KE *S. uvarum* genotype, SuOK04, is closely related to PYCC 6860, a strain previously isolated from an oak tree on Hornby Island in British Columbia (Almeida et al., 2014). Two OO *S. uvarum* strains (SuOK08 and SuOK09) and three NP *S. uvarum* strains (SuOK05, SuOK06, and SuOK07) are more closely related to each other than between their respective sub-regions. By contrast, *S. uvarum* strains isolated from KE (SuOK01, SuOK2, and SuOK04) are distantly related to each other. For example, SuOK01 is more closely related to two *S. uvarum* strains isolated from New Zealand (A1 and A9) (Zhang et al., 2015).

### Flavonoid Composition of Pinot Noir Grape Berries From OV Sub-Regions

In the 2017 vintage, in addition to performing 6 spontaneous fermentations from each of 13 OV vineyards, we also sampled the Pinot Noir berries from each vineyard to determine how grape berry weight and metabolites may differ in the 3 OV sub-regions. In particular, we focused on the three major flavonoid classes: anthocyanins, flavonols, and tannins. We observed that the berry weight as well as the number of seeds per berry were affected by the sub-region (Supplementary Table S11). The average berry weight and seed number were higher in NP than OO and KE. The other parameters analyzed – skin weight, seed weight, skin to berry ratio, seed to berry ratio, and TSS – did not vary among the three OV sub-regions. Importantly, the TSS were not significantly different among all three sub-regions, ensuring that we could accurately compare the flavonoid composition in the Pinot Noir berries.

**FIGURE 6:**
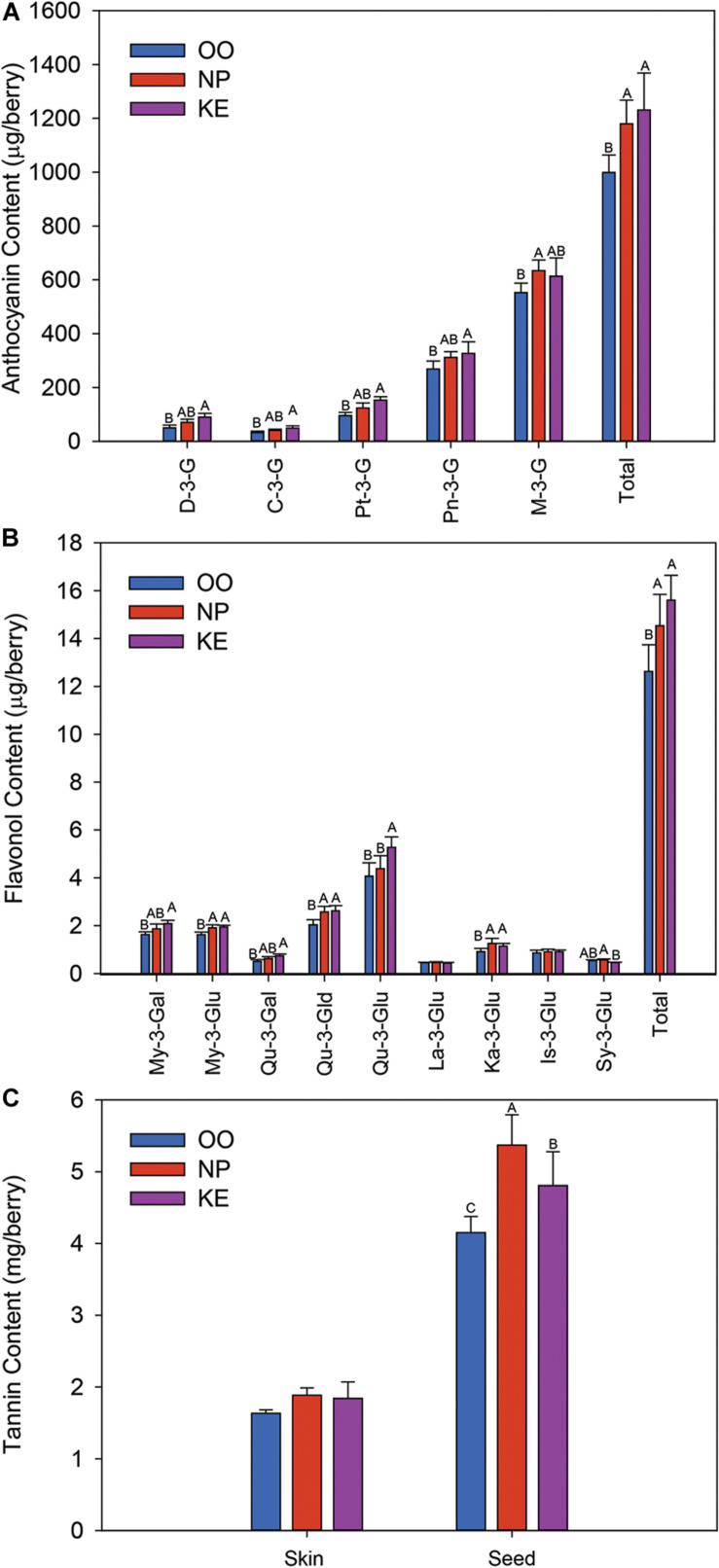
Anthocyanin **(A)**, flavonol **(B)**, and tannin **(C)** content (amount per berry) of Pinot Noir grapes from three sub-regions of the OV (OO, blue; NP, red; and KE, purple). D-3-G, delphinidin-3-glucoside; C-3-G, cyanidin-3-glucoside; Pt-3-G, petunidin-3-glucoside; Pn-3-G, peonidin-3-glucoside; M-3-G, malvidin-3-glucoside for anthocyanins **(A)**; and My-3-Gal, myricetin-3-galactoside; My-3-Glu, myricetin-3-glucoside; Qu-3-Gal, quercetin-3-galactoside; Qu-3-Gld, quercetin-3-glucuronide; Qu-3-Glu, quercetin-3-glucoside; La-3-Glu, laricitrin-3-glucoside; Ka-3-Glu, kaempferol-3-glucoside; Is-3-Glu, isorahamnetin-3-glucoside; Sy-3-Glu, syringetin-3-glucoside for flavonols **(B)**. Error bars indicate the standard error within each sub-region. An ANOVA was performed to test the effect of the sub-regions with vineyards considered as nested factors within sub-regions. Different letters indicate significant differences (*p* < 0.05) between sub-regions according to a Tukey’s HSD test.

Five anthocyanins were identified and quantified in all the Pinot Noir grape samples collected; the monoglucosides of delphinidin (D-3-G), cyanidin (C-3-G), petunidin (Pt-3-G), peonidin (Pn-3-G), and malvidin (M-3-G) (Figure 6A). M-3-G was the most abundant anthocyanin while C-3-G was the least abundant anthocyanin. The content (expressed as μg/berry) of all anthocyanins except M-3-G was higher in KE than OO. NP had intermediate levels of all anthocyanins except M-3-G which was higher in NP than OO. The content of total anthocyanins was higher in KE and NP than OO. The concentrations (expressed as μg/g berry) of D-3-G, C-3-G and Pt-3-G were greater in KE than OO berries, while Pn-3-G and M-3-G concentrations were not affected by the sub-region (Supplementary Figure S1). The concentration of total anthocyanins was higher in KE than OO (Supplementary Figure S1A).

Nine flavonols were identified in Pinot Noir grapes collected from the three OV sub-regions (Figure 6B). These compounds were the glucosides of myricetin (My-3-Glu), quercetin (Qu-3-Glu), laricitrin (La-3-Glu), kaempferol (Ka-3-Glu), isorhamnetin (Is-3-Glu), and syringetin (Sy-3-Glu), the galactosides of myricetin (My-3-Gal) and quercetin (Qu-3-Gal), and the glucuronide of quercetin (Qu-3-Gld). Qu-3-Glu was the most abundant flavonol compound in the Pinot Noir berries while La-3-Glu and Sy-3-Glu were the least abundant flavonols identified (Figure 6B). The content (expressed as μg/berry) of all quercetin conjugates was higher in KE than OO berries. Qu-3-Glu was also higher in KE than NP berries while Qu-3-Gld was as high in NP as KE berries. The content of myricetin conjugates was also higher in KE than OO berries (Figure 6B). My-3-Gal content was intermediate in NP berries whereas My-3-Glu content was as high in NP as KE berries. Similar results were observed when the flavonols were reported as a concentration (expressed as μg/g berry). The concentration of most flavonols were higher in KE than OO and NP generally had intermediate levels (Supplementary Figure S1B). The content of total flavonols was higher in KE and NP than OO berries (Figure 6B) and the concentration was higher in KE than OO berries, while NP berries had intermediate levels (Supplementary Figure S1B).

Unlike the anthocyanin and flavonol data, the skin tannin content and concentration of Pinot Noir grapes did not vary among the three OV sub-regions (Figure 6C and Supplementary Figure S1C). However, the seed tannin content (expressed as mg/berry) and concentration (expressed as mg/g berry) were the highest in NP berries and the lowest in OO berries, and at intermediate levels in KE berries (Figure 6C and Supplementary Figure S1C).

**FIGURE 7:**
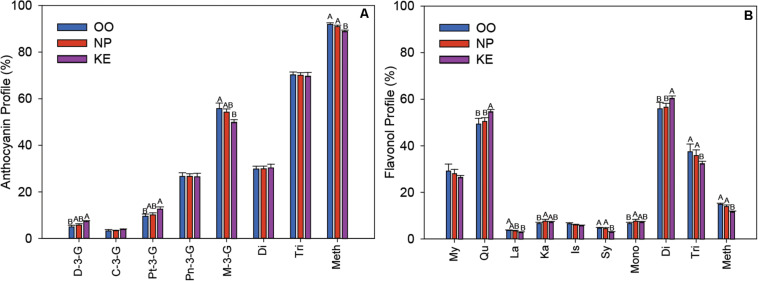
Relative abundance of individual anthocyanins and anthocyanin fractions **(A)** and individual flavonols and flavonol fractions **(B)** in the three sub-regions of the OV (OO, blue; NP, red; and KE, purple). C, cyanidin; Pn, peonidin; D, delphinidin; Pt, petunidin; M, malvidin; Di, di-substituted; Tri, tri-substituted; Meth, methoxylated for anthocyanins **(A)**; and My, myricetin; Qu, quercetin; La, laricitrin; Ka, kaempferol; Is, isorhamnetin; Sy, syringetin; Mono, mono-substituted; Di, di-substituted; Tri, tri-substituted; Meth, methoxylated for flavonols **(B)**. Error bars indicate standard error within each sub-region. An ANOVA was performed to test the effect of the sub-regions with vineyards considered as nested factors within sub-regions. Different letters indicate significant differences (*p* < 0.05) between sub-regions according to a Tukey’s HSD test.

The profile of the relative abundance of each anthocyanin was plotted as a percentage of the total amount of anthocyanins in the Pinot Noir grape samples from each sub-region (Figure 7A). The major anthocyanin was M-3-G in all three sub-regions, however, the relative abundance of M-3-G was higher in OO (55.79 ± 2.36%) than KE (49.89 ± 1.13%) berries. In contrast, the relative abundances of both D-3-G and Pt-3-G were higher in KE than OO berries. We also grouped the anthocyanins into di-substituted, which refers to two substituted sites (e.g., hydroxylation and methoxylation) at the B-ring (Cy-3-G and Pn-3-G), and tri-substituted, which refers to three substituted sites at the B-ring (D-3-G, Pt-3-G, and M-3-G), groups (Bakowska-Barczak, 2005). We found that the relative abundance of di-substituted and tri-substituted anthocyanins did not change among OV sub-regions (Figure 7A). We also considered the level of methoxylation at the B-ring of the anthocyanins. Interestingly, we found that the relative abundance of methoxylated anthocyanins (Pt-3-G, Pn-3-G, and M-3-G) was higher in OO and NP than KE berries (Figure 7A).

The six aglycones of flavonols, detected in the various glycoside forms, were myricetin, quercetin, laricitrin, kaempferol, isorhamnetin, and syringetin and their relative abundances were plotted as a percentage of total flavonols per OV sub-region (Figure 7B). Quercetin was the most abundant flavonol with a higher relative abundance in KE (54.68 ± 0.91) than NP (50.51 ± 1.58%), and OO (49.40 ± 2.43%) berries. The relative abundance of laricitrin was higher in OO than KE berries whereas syringetin was higher in OO and NP than KE berries. As with the anthocyanins, we compared groups of mono-, di-, and tri-substituted flavonols across the OV sub-regions. We found that the relative abundance of mono-substituted flavonols (kaempferol) was higher in NP than OO berries, di-substituted flavonols (isorhamnetin and quercetin) was higher in KE than NP and OO berries, and tri-substituted flavonols (myricetin, laricitrin, and syringetin) was higher in OO and NP than KE berries (Figure 7B). Similar to what we discovered for anthocyanins, the relative abundance of methoxylated flavonols (laricitrin, isorhamnetin, and syringetin) was higher in OO and NP than KE Pinot Noir berries (Figure 7B).

## Discussion

In this study we surveyed 100 km of a wine region in British Columbia, Canada for yeast populations and flavonoids associated with Pinot Noir berries. We sampled from 13 vineyards which were grouped into the northern (KE), central (NP), and southernmost (OO) sub-regions of the OV. We performed spontaneous fermentations using grapes sampled from 13 vineyards for two consecutive vintages for a total of 156 spontaneous fermentations. Fewer than 50% of the grape must samples achieved two-thirds sugar depletion within 40 days of fermentation, with little consistency between vintages (Supplementary Table S7). In part, this may be due to our method of grape sampling, where grapes were taken from six discrete vineyard sections, and also due to the relatively low abundance (0.1%) of *S. cerevisiae* in the fungal microflora of a grape berry (Mortimer and Polsinelli, 1999; Tofalo et al., 2013). Our results reflect those in previous studies where unpredictability is associated with spontaneous fermentation, including a dynamic vineyard yeast population between vintages and geographical locations (Schuller et al., 2012; Knight and Goddard, 2015; Rantsiou et al., 2017).

The number of fermentations reaching two-thirds sugar depletion from NP in both vintages (8) was markedly lower than KE (28) and OO (26) (Supplementary Table S7). The fewer wine yeasts isolated from NP may be explained by various factors including the proximity to winery facilities, wind and weather patterns. Two NP vineyards (#5 and #8) are situated over 1km from the nearest known winery, while two others (#6 and #9) are located at least 100 m uphill of wineries, against prevailing wind conditions (Figure 1). NP also has the highest cumulative precipitation and lowest cumulative GDD among the 3 OV sub-regions in both vintages (Government of Canada, 2020). Rain and cool temperatures may discourage the development of *S. cerevisiae* on fruit as well as the presence of other biological vectors (birds and insects) that are involved in the dispersal of wine yeasts (Goddard and Greig, 2015). While very few strains were shared between sub-regions, the most distant sub-regions (OO and KE) had the most strains in common (Figure 2A). Although NP is situated between KE and OO, these two latter sub-regions are much larger in size than NP and may have greater trafficking of equipment and personnel between them. OO is better suited to grow later ripening red grape cultivars due to its warmer climate, and many KE wineries may source grapes from or farm vineyards in this sub-region.

The isolation of either *S. cerevisiae*, *S. uvarum*, or both species from each vineyard and each vintage had no clear pattern except that *S. uvarum* was only isolated in the 2017 vintage (Figure 1). This result may correspond to the dynamic composition of vineyard-associated microbiota, which varies between vintages due to a variety of factors (Bokulich et al., 2014). In 2017, the number of *S. cerevisiae* isolates was over sevenfold higher than for *S. uvarum* (1,176 vs. 164 isolates, Supplementary Table S7). *S. uvarum* has lower ethanol and temperature tolerance that may result in a stuck fermentation at 24°C (Masneuf-Pomarede et al., 2010). Furthermore, due to its crytolerant traits, *S. uvarum* has typically been identified in white wine fermentations at lower temperatures (∼15°C) (Demuyter et al., 2004; Masneuf-Pomarede et al., 2010; Lopez-Malo et al., 2013; Knight and Goddard, 2015). Therefore, it was surprising to isolate *S. uvarum* from a Pinot Noir spontaneous fermentation carried out at ambient temperature (25°C) that may not favor the growth of *S. uvarum.* Moreover, although *S. uvarum* can out-compete *S. cerevisiae* in mixed culture fermentations with *S. cerevisiae* at 12°C, *S. uvarum* was isolated as a minority species in fermentations at 25°C in this study, which is expected as *S. cerevisiae* has a better fermentative competitiveness and ethanol tolerance at 25°C (Alonso-Del-Real et al., 2017; Su et al., 2019; Morgan et al., 2020). *S. paradoxus* was only identified from one KE vineyard in a single fermentation that also contained *S. cerevisiae* (Figure 1). We isolated 28 colonies of *S. paradoxus* but did not carry out strain genotyping, therefore the number of strains is unknown (Supplementary Table S7). Although *S. paradoxus* is typically more likely to be isolated from wild environments, multiple studies have identified *S. paradoxus* in vineyards and spontaneous fermentations (Redzepovic et al., 2002; Valero et al., 2007; Hyma and Fay, 2013; Sipiczki, 2016; Vaudano et al., 2019).

As found in other wine regions, *S. cerevisiae* populations differed among the three sub-regions sampled (Gayevskiy and Goddard, 2012; Schuller et al., 2012; Knight and Goddard, 2015). Among the 103 identified *S. cerevisiae* strains, there were only 4 strains identified in more than one sub-region and no strains identified in all three sub-regions (Figure 2). AMOVA results further indicate that *S. cerevisiae* populations are genetically dissimilar between OO and KE sub-regions, which suggests that geography is a driver of population structure within the OV wine region (Table 1). Variation was higher amongst vineyards within the same sub-region and vintage, rather than sub-regions themselves, highlighting the high heterogeneity of *S. cerevisiae* distribution between and within vineyards (Table 1). Viticulture practices among the vineyards sampled in this study were variable, with several vineyards employing organic practices. Viticulture management techniques can influence the fungal biota on fruit, which may in turn impact the diversity of *S. cerevisiae* strains in the vineyard environment (Setati et al., 2012; Morrison-Whittle et al., 2017). Our study also systematically profiled *S. cerevisiae* populations in the same sections of the same vineyards over 2 years, enabling a direct comparison of populations between vintages. Interestingly, while very few MLGs were isolated from both vintages, no significant difference was found between 2016 and 2017 *S. cerevisiae* populations (Table 1). It is reasonable to expect that yeast populations may remain similar over multiple years, as *S. cerevisiae* can reside in soil and in environments proximate to vineyards, which act as reservoirs for the yeast during winter (Knight and Goddard, 2016; Sipiczki, 2016; Gonzalez et al., 2020).

Even though the grapes we sampled never entered a winery, we still identified a relatively high proportion of commercial MLGs (40.7%) in the dataset (Figure 2). Our previous work in three closely situated OV vineyards also found commercial yeast dissemination into the vineyards and a wider regional survey in Italy similarly found a high number of commercial *S. cerevisiae* strains in vineyards across multiple sub-regions (Martiniuk et al., 2016; Viel et al., 2017). The occurrence of commercial yeast genotypes within the different sub-regions has a weakening effect on the differentiation of sub-regional yeast populations as observed in AMOVA results, which is consistent with a previous study (Viel et al., 2017). It is very likely that many of the commercial *S. cerevisiae* strains identified in this study originated from the wineries that source grapes from the vineyards we sampled. These strains could be introduced into the vineyard if the winery facility is in close proximity (Valero et al., 2005). Using composted winery waste (e.g., pomace and lees) as fertilizer may also introduce cellar yeast into the vineyard. While we do not have composting data for all participating vineyards, at least three vineyards (#1, #7, and #8) composted winery waste in one or both years of this study, however, commercial strains were only isolated from one of these vineyards. It is also possible that other biological vectors such as insects, birds or human activity may have transported these and other strains shared between sub-regions (Goddard et al., 2010; Francesca et al., 2012). Curiously, we also identified 2 unknown strains isolated from NP (OK11 and OK12) that are related to commercial beer yeast strains (Figure 4). OK11 and OK12 may have originated from a brewery located within NP and were introduced by human or other vectors into the vineyard.

InStruct results indicate that there were two clusters of *S. cerevisiae* strains (K8 and K9) that appear unassociated with commercial strains isolated from the various vineyards (Figure 3). The phylogenetic network of unknown and commercial strains compiled for this study further indicates that there are sub-populations of *S. cerevisiae* strains that are genetically distinct from commercial strains (Figure 4). There is an intimate genetic association between global *S. cerevisiae* wine strains and *S. cerevisiae* wine strain populations in Europe, which implies that European wine strains migrated around the globe and became separate sub-populations (Borneman et al., 2016; Gayevskiy et al., 2016; Peter et al., 2018). Further studies characterizing the genomic similarity between unknown OV strains and European wine strains using whole genome sequencing will be necessary to understand the origin of *S. cerevisiae* strains identified in OV.

None of the 9 identified *S. uvarum* genotypes were isolated from multiple sub-regions. This may indicate that *S. uvarum* genotypes were exclusively associated to a sub-region, although because fermentation conditions were not optimized for *S. uvarum* enrichment, it is difficult to conclude this. The majority of the *S. uvarum* genotypes appear closely related to strains previously isolated from the OV, with the exception of SuOK4, which is closely related to PYCC6860 (Figure 5). Interestingly, PYCC6860 was previously isolated from an oak tree on Hornby Island off the coast of Vancouver, British Columbia which is ∼600 km away from the OV (Almeida et al., 2014). The ancestry of *S. uvarum* strains SuOK01 and SuOK02, which appear more distantly related to the reference *S. uvarum* strains, are unknown. *S. uvarum* strains associated with European wine fermentation have prevalent and extensive introgressions from *Saccharomyces eubayanus* whereas strains isolated from the environment, such as PYCC6860, do not (Almeida et al., 2014). Two of our *S. uvarum* strains (SuOK03 and SuOK08) were identical to strains (MLG13 and OV13-11, respectively) that were isolated from industrial spontaneous fermentations conducted in the OV in previous vintages [(McCarthy et al., Unpublished) and unpublished data from this group]. The discovery of these previously isolated *S. uvarum* strains in KE and OO suggests that these strains may be prevalent in OV vineyards, or that they were introduced from the winery via a biological vector.

In the 2017 vintage, we combined our yeast population study with a metabolite analysis of Pinot Noir berries from the same 13 vineyards. Berries collected from all three OV sub-regions contained similar amounts of TSS indicating that grape samples were generally collected at similar developmental stages and ripeness among sub-regions (Supplementary Table S11). Therefore, we can exclude ripening effects as a factor influencing our results. Pinot Noir berry weight and seed number were both higher in the NP sub-region than OO and KE which is probably related to higher number of seeds per berry, which was also higher in NP than OO and KE (Supplementary Table S11) (Keller, 2010).

Pinot Noir grapes sampled from OO had lower anthocyanin content than NP and KE (Figure 6A). Changes in anthocyanin accumulation are likely associated with sub-regional differences in anthocyanin biosynthesis and/or degradation rather than to variation in berry size (Mori et al., 2007). Smaller berry size often results in a higher skin:berry ratio and can promote the concentration of anthocyanins and other skin phenolics (Roby et al., 2004; Wong et al., 2016). However, in this study we observed no differences in skin:berry ratio among sub-regions (Supplementary Table S11). In general, anthocyanin biosynthesis is affected by biotic and abiotic factors [reviewed in Teixeira et al. (2013)]. Temperature is known to strongly affect anthocyanin accumulation in grapes (Spayd et al., 2002; Yamane et al., 2006; Mori et al., 2007; Nicholas et al., 2011). At moderate growing temperatures (i.e., 20–25°C) anthocyanin accumulation is promoted, while at relatively high temperatures (30–35°C) anthocyanin accumulation is reduced, possibly because of a lower biosynthesis and/or higher rate of degradation (Yamane et al., 2006; Mori et al., 2007; Artem et al., 2016). Therefore, the temperature differences in the OV sub-regions may have contributed to the observed differences in anthocyanins (Figure 6A). Among these 3 sub-regions, active heat summation was highest in OO, with 1,513 GDD, followed by KE, with 1,263 GDD, and lowest in NP, with 1,157 GDD. Furthermore, the number of days with the maximum temperature reaching 35°C was greater in OO (12 days) than KE (7 days) and NP (2 days). Consistent with the previous studies reported above, the lowest levels of anthocyanins were observed in the warmest region (OO).

Aside from temperature, water availability is another factor affecting anthocyanin accumulation because water deficit generally promotes anthocyanin accumulation (Matthews and Anderson, 1989; Castellarin et al., 2007; Savoi et al., 2017). The OV sub-regions had also different cumulative precipitation from April 01 to October 31, 2017. The highest was recorded in NP (198 mm), followed by OO (140 mm), and the lowest in KE (121 mm). Moreover, OO soils are characterized by a lower water holding capacity than NP and KE soils (British Columbia Wine Institute, 2020). However, all the sampled vineyards were irrigated in order to avoid water deficit events. Therefore, we speculate that precipitation and water availability in general had no effect or a limited effect in determining the anthocyanin differences observed among the OV sub-regions.

Similar to the concentration of anthocyanins, flavonol levels varied among the three OV sub-regions (Figure 6B). Previous studies have demonstrated that temperature variation and water availability have little or inconsistent effects on flavonol accumulation (Price et al., 1995; Spayd et al., 2002; Downey et al., 2004; Azuma et al., 2012). However, a recent study from our group indicates that, as for anthocyanins, high temperatures (i.e., 30–35°C) impair flavonol accumulation in the berry which is consistent with our observations here that the lowest level of flavonols is in OO, the warmest region (Yan et al., 2020). Studies have shown that sunlight intensity, and particularly the intensity of UV light, positively correlates with flavonol levels in grapes (Price et al., 1995; Spayd et al., 2002; Azuma et al., 2012; Martinez-Luscher et al., 2014a, b; Del-Castillo-Alonso et al., 2016). In this study, radiation levels were not available and a correlation analysis could not be performed.

Seed tannin, but not skin tannin content was affected by the sub-region and the seed tannin data was consistent with the number of seeds per berry (Figure 6C and Supplementary Table S11). OO had both a lower seed number per berry and lower seed tannin content than NP. This is consistent with previous results indicating that seed tannin content varies with the seed number (Harbertson et al., 2002). Previous studies on the association of grape tannin content with *terroir* have yielded inconsistent results (Ferndandez-Marin et al., 2013; Artem et al., 2016). The effect of environmental factors such as temperature and water availability on tannins still remains unclear as contrasting results have been reported among studies indicating that the grape variety, as well as the interaction among various environmental factors affect tannin content (Cohen et al., 2008; Nicholas et al., 2011; Zarrouk et al., 2012; Genebra et al., 2014; Kyraleou et al., 2017). Based on our study, high temperature (see GDD for OO above) is likely to reduce seed tannin accumulation in Pinot Noir, while a low temperature (see GDD for NP above) is likely to enhance seed tannin accumulation.

Although the proportion of several anthocyanins and flavonols were significantly different in OV sub-regions, there was little consistency among the various compounds. The major anthocyanin accumulated in Pinot Noir berries, M-3-G, was proportionally higher in OO than KE berries and yet, KE and NP berries had the highest total anthocyanin levels (Figures 6A, 7A). Anthocyanins with a higher number of methoxylated groups present in the B-ring, such as M-3-G, are more stable (Yang et al., 2018). Grape berries grown at high temperature (30–35°C) normally have a higher relative abundance of methoxylated anthocyanins and flavonols in grape skins (Mori et al., 2007; Zhu et al., 2017; Yan et al., 2020). The higher proportion of methoxylated anthocyanins in OO than KE berries could be linked to the higher temperatures in OO compared to KE (Figure 7A, see GDD for these two regions above). Higher temperatures may favor the synthesis of the more stable methoxylated anthocyanins or, alternatively, may favor the degradation of the non-methoxylated anthocyanins, which in either scenario would cause a relative increase of the methoxylated fraction.

The relative abundance of mono-, di-, tri-substituted flavonols varied by sub-region. We found that the coolest region, KE, had an increase in di-substituted but a decrease in tri-substituted flavonols when compared to OO and NP (Figure 7B). Our data is consistent with our recent study of Merlot berries that demonstrated similar effects of di- versus tri-substituted flavonols when low temperatures were compared to high temperatures (Yan et al., 2020). Moreover, Pastore et al. (2017) reported a higher proportion of di-substituted flavonols with increased light exposure of berries. The changes observed in our study are probably driven by the increase in the quercetin conjugates observed in KE berries (Figure 6B). Quercetin, a di-substituted flavonol, is the major flavonol produced in Pinot Noir berries and its variable levels in the OV sub-regions might be the major determinant of the shifts in flavonol profile.

At present, we do not have sufficient data to determine if there is any correlation between the *Saccharomyces* strains isolated from OV sub-regions and the flavonoid profiles presented here. It has been shown that grape cultivars may drive *S. cerevisiae* population structure, suggesting there may be adaptation or preference of yeasts to certain grape cultivars (Schuller et al., 2012). *S. cerevisiae* strains have variable effects on final phenolic composition in red wines, which indicates the potential for interaction between regional yeast populations and grape phenolic profiles that is reflected in the finished wine (Carew et al., 2013). Further research is required to determine how regional-specific wine yeast strains and the flavonoid profile of Pinot Noir grapes affect the quality of wine production in the OV.

## Data Availability Statement

All datasets generated for this study are included in the article/Supplementary Material.

## Author Contributions

EC collected samples, performed and analyzed *Saccharomyces* microsatellite and polyphenolic data for the 2017 vintage, and contributed to the writing of the manuscript. JM collected samples, designed experiments, performed *Saccharomyces* microsatellite and population analysis for 2016 and 2017 vintages, and contributed to the writing of the manuscript. JH monitored and sampled fermentations and performed commercial *S. cerevisiae* microsatellite analyses. GM collected samples, monitored and sampled fermentations, and performed *Saccharomyces* microsatellite analyses. SC collected samples, analyzed polyphenolic data, and contributed to writing of the manuscript. VM analyzed *Saccharomyces* population data and wrote the manuscript. All authors contributed to the article and approved the submitted version.

## Conflict of Interest

The authors declare that the research was conducted in the absence of any commercial or financial relationships that could be construed as a potential conflict of interest.
