# Supplementary figures and images for: Characterization of Sub-Regional Variation in Saccharomyces Populations and Grape Phenolic Composition in Pinot Noir Vineyards of a Canadian Wine Region
Source: Front Genet. 2020 Aug 31;11:908. doi: 10.3389/fgene.2020.00908 (PMC7489054; doi:10.3389/fgene.2020.00908)

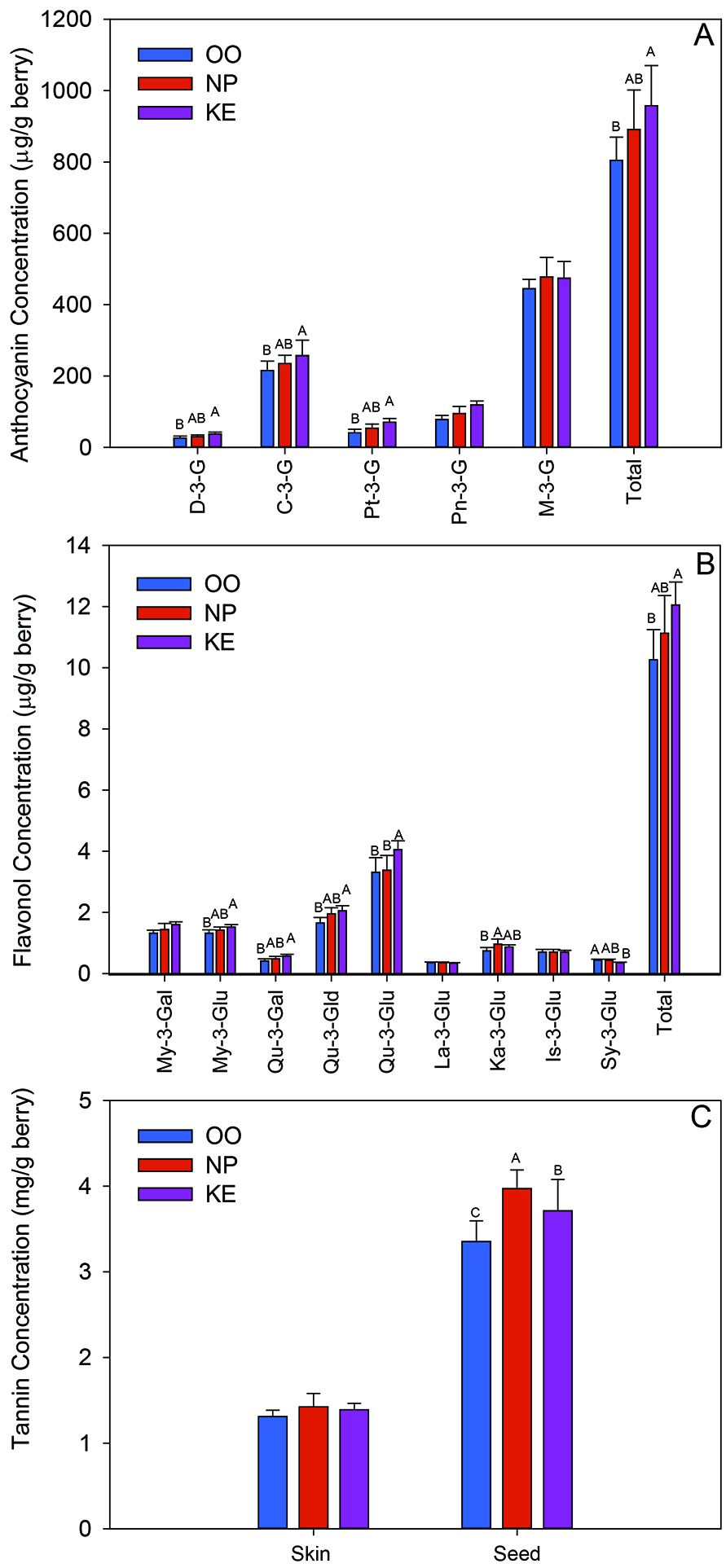

Supplement: Supplementary file 1 [file Image_1.tif]
