# Supplementary material for: Characterization of Sub-Regional Variation in Saccharomyces Populations and Grape Phenolic Composition in Pinot Noir Vineyards of a Canadian Wine Region
Source: Front Genet. 2020 Aug 31;11:908. doi: 10.3389/fgene.2020.00908 (PMC7489054; doi:10.3389/fgene.2020.00908)
Supplement: Supplementary file 3 [file Table_2.DOCX]

**Table S2.** STR Primers used for *S. cerevisiae* Microsatellite Analysis

| Locus | Chromosome | Tag | SSR | Alleles^a^ | Size range (bp) |
| --- | --- | --- | --- | --- | --- |
| YGL139W (C3)* | VII | FAM | CAA | 18 | 94 – 158 |
| YLR177W (YLR) | XII | PET | CAG | 13 | 80 – 160 |
| YFR028C (C5) | VI | VIC | GT | 34 | 119 – 228 |
| YGL014W (C8) | VII | NED | TAA | 10 | 122 – 160 |
| YOL109W (C4) | XV | VIC | TAA, TAG | 26 | 230 – 373 |
| YML091C (O91C) | XIII | NED | AAT | 31 | 218 – 331 |
| YOR267c (SCY) | XV | PET | CAA | 27 | 258 – 390 |
| YLL049W (AT4) | XII | FAM | TA | 15 | 270 – 305 |
| YDR160W (SCAAT3) | IV | NED | AAT | 21 | 345 – 499 |
| YPL009C (009C) | XVI | FAM | CTT | 21 | 385 – 456 |

^*^Abbreviation written in parentheses is the common name of the loci. ^a^The total number of unique alleles in the database.
