# Supplementary material for: Characterization of Sub-Regional Variation in Saccharomyces Populations and Grape Phenolic Composition in Pinot Noir Vineyards of a Canadian Wine Region
Source: Front Genet. 2020 Aug 31;11:908. doi: 10.3389/fgene.2020.00908 (PMC7489054; doi:10.3389/fgene.2020.00908)
Supplement: Supplementary file 4 [file Table_3.DOCX]

**Table S3.** STR Primers used for S. uvarum Microsatellite Analysis

| Locus | Chromosome | Tag | SSR | Alleles^a^ | Size range  (bp) |
| --- | --- | --- | --- | --- | --- |
| NB1 | X | FAM | ATG | 5 | 193 – 212 |
| NB4 | X | FAM | TGT | 4 | 334 – 350 |
| NB8 | XVI | FAM | TGT | 5 | 415 – 450 |
| NB9 | XV | FAM | AT | 6 | 111 – 121 |
| SuARS409 (L1)* | X | FAM | GT | 3 | 163 – 167 |
| SuYBR049c (L2) | II | FAM | ATT | 8 | 285 – 306 |
| SuYKL017c (L3) | XI | FAM | TA | 4 | 219 – 229 |
| SuYKR045c (L4) | XI | VIC | CTG | 9 | 293 – 334 |
| SuHTZ1PLB3 (L7) | XII | NED | TC | 4 | 261 – 271 |
| SuYHR102W (L8) | VIII | NED | GTT | 11 | 202 – 254 |
| SuYIL130W (L9) | IX | VIC | ATT | 19 | 173 – 290 |

^*^Abbreviation written in parentheses is the common name of the loci. ^a^Number of unique alleles in the database and generated from this project.
