# Supplementary material for: Characterization of Sub-Regional Variation in Saccharomyces Populations and Grape Phenolic Composition in Pinot Noir Vineyards of a Canadian Wine Region
Source: Front Genet. 2020 Aug 31;11:908. doi: 10.3389/fgene.2020.00908 (PMC7489054; doi:10.3389/fgene.2020.00908)
Supplement: Supplementary file 5 [file Table_4.DOCX]

**Table S4.** S. cerevisiae Microsatellite Primer Mix Sequences

| Locus | Volume^a^ (µl) | Primer Sequence |
| --- | --- | --- |
| C3 F^b^ | 9.5 | GTGTCTCTTTTTATTTACGAGCGGGCCAT |
| C3 R^c^ | 9.5 | AAATCTCATGCCTGTGAGGGGTAT |
| YLR F | 5.4 | CTTAAACAACAGCTCCCAAA |
| YLR R | 5.4 | ATGAATCAGCGCATCAGAAAT |
| C5 F | 9.5 | GTGTCTTGACACAATAGCAATGGCCTTCA |
| C5 R | 9.5 | GCAAGCGACTAGAACAACAATCACA |
| C8 F | 18 | GTGTCTCAGGTCGTTCTAACGTTGGTAAAATG |
| C8 R | 18 | GCTGTTGCTGTTGGTAGCATTACTGT |
| C4 F | 48.4 | GTGTCTAGGAGAAAAATGCTGTTTATTCTGACC |
| C4 R | 48.4 | TTTTCCTCCGGGACGTGAAATA |
| O91 F | 0 | GTGTCTAAGCCTCTTCAAGCATGAC |
| O91 R | 30 | GTGTCTGGACAATTTTGCCACCTTA |
| SCY F | 8 | TACTAACGTCAACACTGCTGCCAA |
| SCY R | 8 | GGATCTACTTGCAGTATACGGG |
| AT4 F | 20 | GCAACATAATGATTTTGAGGT |
| AT4 R | 20 | GTGTCTTGTGTGAGCATAGTGGAGAA |
| SCAAT3 F | 90 | GTGTCTGAGGAGGGAAATGGACAG |
| SCAAT R | 90 | GCCTGAAGATGCTTTTAG |
| 009C F | 20 | GTGTCTGGGTTTTGGATTTTTATGGA |
| 009C R | 20 | GTGTCTTTCAATTTTCCTCTTTTACCAC |
| Milli-Q water | 1464.4 |  |

^a^The volume is for 2000 reactions and concentrations of the primers were 100 µM. ^b^F= forward primer; ^c^R= reverse primer
