# Supplementary material for: Characterization of Sub-Regional Variation in Saccharomyces Populations and Grape Phenolic Composition in Pinot Noir Vineyards of a Canadian Wine Region
Source: Front Genet. 2020 Aug 31;11:908. doi: 10.3389/fgene.2020.00908 (PMC7489054; doi:10.3389/fgene.2020.00908)
Supplement: Supplementary file 6 [file Table_5.DOCX]

**Table S5.** S. uvarum Microsatellite Primer Mix Sequences

| Locus | Volume^a^ (µl) | Primer Sequence |
| --- | --- | --- |
| NB1 F^b^ | 20 | GTGCTCCATGGACTTGTATGAAGCAA |
| NB1 R^c^ | 20 | GTTCGTTACCTTCAGTGCTC |
| NB4 F | 60 | GTGCTCGACATTGTAAAAGCACAGCA |
| NB4 R | 60 | ACGGGGCTTCTCTAGATATT |
| NB8 F | 80 | GTGCTCTGCATGAAAGATTGTAAAGG |
| NB8 R | 80 | TCCACAACGATATCAAGACA |
| NB9 F | 80 | GTGCTCAAACAAGAAACTGTGGTCGT |
| NB9 R | 80 | TGCTTTAATTTCAAGAAACA |
| L1 F | 60 | CGTGTTGAAGACATAATTG |
| L1 R | 60 | AATCTGAACGACAGGAAT |
| L2 F | 160 | TGCCCTTCTTATTCTTGT |
| L2 R | 160 | GAAAATATCAACGCATTAAA |
| L3 F | 130 | GTATGCATCACTATTTTTCG |
| L3 R | 130 | AATTTGGTAATTTGAATGTG |
| L4 F | 60 | GGACACTAGAGTTCGTCTCG |
| L4 R | 60 | GCCACCACTATCAGTTCG |
| L7 F | 50 | GTAGAATTCACCACAGGTC |
| L7 R | 50 | CCGTATATAAAACAGCACTT |
| L8 F | 50 | CACGGCAATCAGCACATTT |
| L8 R | 50 | TGAAGTTTCATCATCGGCAA |
| L9 F | 160 | AAAAAGCAACCTTAAAAGCAACA |
| L9 R | 160 | CTTTACGTAGGCTCATGGCA |
| Milli-Q water | 180 |  |

^a^The volume is for 2000 reactions. The concentrations of the primers were 10 µM for all primers except L3, L4, L7, L8 and L9, which were 15 uM. ^b^F= forward primer; ^c^R= reverse primer
