# Supplementary material for: Characterization of Sub-Regional Variation in Saccharomyces Populations and Grape Phenolic Composition in Pinot Noir Vineyards of a Canadian Wine Region
Source: Front Genet. 2020 Aug 31;11:908. doi: 10.3389/fgene.2020.00908 (PMC7489054; doi:10.3389/fgene.2020.00908)
Supplement: Supplementary file 7 [file Table_6.DOCX]

**Table S6.** List of Previously Isolated S. uvarum Strains

| Strain | Locality/Country of origin |
| --- | --- |
| CBS7001 | Spain^a^ |
| CBS8690 | Moldova^a^ |
| CBS8696 | USA^a^ |
| CBS8711 | France^a^ |
| A1 | New Zealand^b^ |
| A4 | New Zealand^b^ |
| A9 | New Zealand^b^ |
| 7A6 | New Zealand^b^ |
| 7D4 | New Zealand^b^ |
| 8B11 | New Zealand^b^ |
| CBS 395 | Netherlands^b^ |
| PYCC6330 | South America^c^ |
| PYCC6860 | Hornby Island, British Columbia, Canada^c^ |
| PYCC6861 | Hornby Island, British Columbia, Canada^c^ |
| PYCC6862 | Japan^c^ |
| PYCC6871 | Portugal^c^ |
| PYCC6895 | Moldova^c^ |
| PYCC6901 | USA^c^ |
| PYCC6902 | USA^c^ |
| PYCC6994 | France^c^ |
| BMV 58 | Spain (Commercial strain)^d^ |
| OV13-07 | OV wineries, British Columbia, Canada^e^ |
| OV13-34 | OV wineries, British Columbia, Canada^e^ |
| OV13-02 | OV wineries, British Columbia, Canada^e^ |
| OV13-04 | OV wineries, British Columbia, Canada^e^ |
| OV13-11 | OV wineries, British Columbia, Canada^e^ |
| MLG160 | OV wineries, British Columbia, Canada^f^ |
| MLG 13 | OV wineries, British Columbia, Canada^f^ |
| MLG 79 | OV wineries, British Columbia, Canada^f^ |
| MLG 103 | OV wineries, British Columbia, Canada^f^ |
| Su01 | OV wineries, British Columbia, Canada^g^ |

Collection acronyms: CBS: Centraalbureau voor Schimmelcultures, Utrecht, The Netherlands; PYCC: Portuguese Yeast Culture Collection. ^a^(Nguyen and Boekhout, 2017), ^b^(Zhang et al., 2015), ^c^(Almeida et al., 2014), ^d^(Perez-Torrado et al., 2016), ^e^ previous studies from this lab (unpublished), ^f^(McCarthy et al., 2020), ^g^(Morgan et al., 2019)

**REFERENCES**

Almeida, P., Gonçalves, C., Teixeira, S., and Libkind, D. (2014). A Gondwanan imprint on global diversity and domestication of wine and cider yeast Saccharomyces uvarum. *Nature communications* 5(1). doi: 10.1038/ncomms5044.

McCarthy, G.C., Morgan, S.C., Martiniuk, J.T., Newman, B.L., Measday, V., and Durall, D.M. (2020). An indigenous Saccharomyces uvarum population with high genetic diversity dominates uninoculated Chardonnay fermentations at a Canadian winery. *PLOS One, in submission*.

Morgan, S.C., McCarthy, G.C., Watters, B.S., Tantikachornkiat, M., Zigg, I., Cliff, M.A., et al. (2019). Effect of sulfite addition and pied de cuve inoculation on the microbial communities and sensory profiles of Chardonnay wines: dominance of indigenous Saccharomyces uvarum at a commercial winery. *FEMS Yeast Res* 19(5). doi: 10.1093/femsyr/foz049.

Nguyen, H.V., and Boekhout, T. (2017). Characterization of Saccharomyces uvarum (Beijerinck, 1898) and related hybrids: assessment of molecular markers that predict the parent and hybrid genomes and a proposal to name yeast hybrids. *FEMS Yeast Res* 17(2). doi: 10.1093/femsyr/fox014.

Perez-Torrado, R., Oliveira, B.M., Zemancikova, J., Sychrova, H., and Querol, A. (2016). Alternative Glycerol Balance Strategies among Saccharomyces Species in Response to Winemaking Stress. *Front Microbiol* 7**,** 435. doi: 10.3389/fmicb.2016.00435.

Zhang, H.Y., Richards, K.D., Wilson, S., Lee, S.A., Sheehan, H., Roncoroni, M., et al. (2015). Genetic characterization of strains of Saccharomyces uvarum from New Zealand wineries. *Food Microbiology* 46**,** 92-99. doi: 10.1016/j.fm.2014.07.016.
