# Supplementary material for: Characterization of Sub-Regional Variation in Saccharomyces Populations and Grape Phenolic Composition in Pinot Noir Vineyards of a Canadian Wine Region
Source: Front Genet. 2020 Aug 31;11:908. doi: 10.3389/fgene.2020.00908 (PMC7489054; doi:10.3389/fgene.2020.00908)
Supplement: Supplementary file 8 [file Table_7.DOCX]

**Table S7.** Number of fermentations reaching two-thirds sugar depletion and Saccharomyces colonies isolated from vineyards in 2016 and 2017 vintages^a^

| Sub-region | Vineyard | Fermentations reaching two-thirds sugar depletion | |
| --- | --- | --- | --- |
|  |  | 2016 | 2017 |
| Oliver-Osoyoos | 1 | 3/6^b^ (143) | 3/6 (140) |
|  | 2 | 3/6 (133) | 5/6 (236) |
|  | 3 | 3/6 (143) | 0/6 (0) |
|  | 4 | 5/6 (238) | 4/6 (142,48) |
| Naramata-Penticton | 5 | 0/6 (0) | 3/6 (142) |
|  | 6 | 1/6 (41) | 1/6 (48) |
|  | 7 | 0/6 (0) | 2/6 (94) |
|  | 8 | 0/6 (0) | 0/6 (0) |
|  | 9 | 0/6 (0) | 1/6 (0,48) |
| Kelowna | 10 | 6/6 (277) | 6/6 (234, 18, 28)^c^ |
|  | 11 | 5/6 (238) | 2/6 (46,50) |
|  | 12 | 6/6 (284) | 2/6 (94) |
|  | 13 | 1/6 (47) | 0/6 (0) |

^a^ Sugar depletion was calculated by weight loss due to CO_2_ generation. ^b^The fraction indicates the number of fermentations that reached two-thirds sugar depletion out of the six fermentations. ^c^The first, second and third number in the parentheses indicate the number of *S. cerevisiae*, *S. uvarum* and *S. paradoxus* colonies isolated from each vineyard, respectively.
