# Supplementary material for: Characterization of Sub-Regional Variation in Saccharomyces Populations and Grape Phenolic Composition in Pinot Noir Vineyards of a Canadian Wine Region
Source: Front Genet. 2020 Aug 31;11:908. doi: 10.3389/fgene.2020.00908 (PMC7489054; doi:10.3389/fgene.2020.00908)
Supplement: Supplementary file 11 [file Table_10.DOCX]

**Table S10.** ObStruct analysis of *S. cerevisiae* population structure inferred by InStruct

| **Overall R2 for your data:** | | 0.13 |  |  |
| --- | --- | --- | --- | --- |
| **p-value** | | <0.0001 |  |  |
|  | | **OO** | **PN** | **KE** |
| **Pairwise R2:** | **OO** | NA | 0.08 (0.02) | 0.12 (<0.001) |
| **PN** | | 0.08 (0.02) | NA | 0.01 (1) |
| **KE** | | 0.12 (<0.001) | 0.01 (1) | NA |
|  | |  |  |  |
| R2 without predefined population OO | | 0.01 |  |  |
| R2 without predefined population KE | | 0.08 |  |  |
| R2 without predefined population PN | | 0.12 |  |  |
|  | |  |  |  |
| R2 without inferred population 9 | | 0.06 |  |  |
| R2 without inferred population 7 | | 0.13 |  |  |
| R2 without inferred population 3 | | 0.13 |  |  |
| R2 without inferred population 8 | | 0.13 |  |  |
| R2 without inferred population 5 | | 0.13 |  |  |
| R2 without inferred population 4 | | 0.13 |  |  |
| R2 without inferred population 2 | | 0.13 |  |  |
| R2 without inferred population 10 | | 0.13 |  |  |
| R2 without inferred population 1 | | 0.14 |  |  |
| R2 without inferred population 6 | | 0.15 |  |  |
