# Supplementary material for: Characterization of Sub-Regional Variation in Saccharomyces Populations and Grape Phenolic Composition in Pinot Noir Vineyards of a Canadian Wine Region
Source: Front Genet. 2020 Aug 31;11:908. doi: 10.3389/fgene.2020.00908 (PMC7489054; doi:10.3389/fgene.2020.00908)
Supplement: Supplementary file 12 [file Table_11.docx]

**Table S11.** General berry parameters of Pinot Noir grapes from three sub-regions of the OV

|  | OO | NP | KE | |
| --- | --- | --- | --- | --- |
| Berry weight (g) | 1.24±0.03^B^ | 1.36±0.04^A^ | 1.31±0.06A^B^ | |
| Skin Weight/Berry (μg) | 108.27±3.74 | 113.91±3.91 | 108.43±3.46 | |
| Skin/Berry (%) | 8.62±0.15 | 8.56±0.38 | 8.38±0.31 |  |
| Seed Weight/Berry (μg) | 36.61±0.66 | 37.03±0.35 | 38.36±1.68 |  |
| Seed/Berry (%) | 6.41±0.17 | 7.06±0.18 | 6.69±0.25 |  |
| Seed number | 2.17±0.05^B^ | 2.58±0.08^A^ | 2.31±0.11^B^ |  |
| Total Soluble Solids (^o^Brix) | 22.19±0.36 | 21.82±0.33 | 22.54±0.29 |  |

The values are averages among all the vineyards of each sub-region. Abbreviations: OO = Oliver-Osoyoos; NP = Naramata-Penticton; KE = Kelowna. An ANOVA was performed to test the effect of the sub-regions with vineyards considered as nested factors within sub-regions. Different letters indicate significant differences (p < 0.05) between sub-regions accordingly to a Tukey’s HSD test.
